# The effect of depressive symptoms on cognition in patients with fibromyalgia

**DOI:** 10.1371/journal.pone.0200057

**Published:** 2018-07-05

**Authors:** Olga Gelonch, Maite Garolera, Joan Valls, Gerard Castellà, Olalla Varela, Lluís Rosselló, Josep Pifarre

**Affiliations:** 1 Universitat de Lleida, Lleida, Spain; 2 Brain, Cognition and Behavior: Clinical Research, Consorci Sanitari de Terrassa, Terrassa, Spain; 3 Neuropsychology Unit, Hospital de Terrassa, Consorci Sanitari de Terrassa, Terrassa, Spain; 4 Lleida Institute for Biomedical Research, Dr. Pifarré Foundation, IRBLLEDA, Lleida, Spain; 5 Hospital Universitari Arnau de Vilanova, Lleida, Spain; 6 Reumatology Section, Fibromyalgia and Chronic Fatigue Syndrome Unit, GSS-Hospital Universitari de Santa Maria, Lleida, Spain; 7 GSS- Hospital Universitari de Santa Maria, Lleida, Spain; 8 Sant Joan de Deu Terres de Lleida, Lleida, Spain; University of North Carolina at Chapel Hill, UNITED STATES

## Abstract

**Background:**

Fibromyalgia (FM) patients frequently complain of cognitive problems, but it remains unclear whether these cognitive complaints can be attributed to a dysfunction of the central nervous system or if they can be explained by other factors associated with the disease, such as depression, anxiety and sleep dysfunction.

**Methods:**

One hundred and ten patients with FM were compared with thirty-three patients diagnosed with a depressive disorder (DD) and fifty healthy controls (HC). Several measures of attention and executive functions were used to make these comparisons and the patients were also asked to complete questionnaires on depression, anxiety and sleep quality. Univariate analyses of covariance (ANCOVA) were performed to identify and control confounders and multiple linear models were used to examine the effects of fibromyalgia and depression on cognitive measures.

**Results:**

FM and HC differed significantly with respect to depression, anxiety and sleep dysfunction, whereas FM and DD did not differ in terms of symptoms of depression and anxiety. However, FM was associated with a worse quality of sleep than DD. Comparisons of cognitive performance between groups showed that short-term and working memory and inattention measures were only associated with symptoms of depression, whereas selective attention was associated with both depression and fibromyalgia, and processing speed, cognitive flexibility and inhibitory control showed a significant interaction between depression and fibromyalgia. Moreover, cognitive flexibility and inhibition abilities were specifically associated with FM.

**Conclusion:**

FM patients show a cluster of cognitive impairment in the attentional and executive domains, although some of the symptoms observed could be explained by the severity of the symptoms of depression, while others seem to depend on the effects of fibromyalgia. Implications of the findings for the understanding and management of cognitive impairment of FM patients are discussed.

## Introduction

Fibromyalgia is a chronic disorder characterised by the presence of generalised and diffuse pain. It is generally accompanied by somatic symptoms, fatigue, waking unrefreshed, depression and cognitive dysfunction [1]. Although self-reported cognitive disturbances are widely recognised in patients with fibromyalgia, there is no absolute consensus among researchers regarding patient performance with respect to objective cognitive measures [2]. As a result, many studies have evidenced cognitive impairment in patients with fibromyalgia compared with control groups, with fibromyalgia patients mainly exhibiting problems with working memory processes and/or in their attentional and executive domains, as well as in processing speed [3–8]. However, other studies have failed to find any impaired performance in the cognitive functions of these patients [9–12]. A couple of recent meta-analytical reviews undertaken with a chronic pain population have provided evidence of impairment of working memory and also of three other components of executive functions: response inhibition, complex executive function and set shifting [13,14]. Whatever the approach adopted, these authors also highlighted the high risk of bias in the studies included.

Psychiatric symptoms such as depressive and anxious disorders as well as sleep dysfunction have frequently been associated with fibromyalgia [15–18]. It has been hypothesised that cognitive dysfunction may be correlated with these psychiatric comorbidities. Indeed, the presence of depression in patients with fibromyalgia clearly constitutes a potential confounding factor given the strong evidence for neuropsychological impairment in depressed patients, who typically show deficits in attention, memory, psychomotor speed, processing speed and executive function [19–21]. Even so, the role of mood disorders has not always been taken into account in studies of cognitive dysfunction associated with fibromyalgia. Landro et al [12] reported that both depressed patients and fibromyalgia patients showed a similar pattern of deficient memory. This was particularly evident in long-term memory tasks requiring effortful processing, in which their memory was significantly impaired compared to healthy controls. Similarly, Sühr et al [22] found that depression was significantly related to memory performance in their study that compared samples of patients with fibromyalgia, chronic pain disorders and healthy controls; furthermore, these groups were not different in cognitive performance after controlling for fatigue, pain and depression. Nevertheless, a number of other studies found no association between emotional factors and cognitive function in fibromyalgia patients [7,23–25].

Sleep dysfunction is another factor that should be taken into account in studies of cognitive dysfunction in fibromyalgia. Research has confirmed that these patients suffer from poorer sleep quality than the general population and also non-restorative sleep [26–28]. Studies examining cognitive performance in people with sleep disorders have found an association between disturbed sleep or insomnia and reduced working memory. They have also noted significant impairment in attention and episodic memory [29,30]. This symptom has, however, been largely ignored in research into cognitive functions, both in general for patients with chronic pain and more specifically for patients with fibromyalgia [14]. In addition, it should be added that when this variable has been analysed, mixed results have been obtained. To our knowledge, only one study has so far identified a significant association between objective cognitive measures and sleep dysfunction [31]; they were not identified in the others [3,32,33].

The aim of this study was to add new evidence to previous research and to evaluate the association between fibromyalgia and cognitive impairment, particularly in the domains of attention and executive functions, controlling for the effects of depression, anxiety and sleep quality. We also carried out a study with a special focus: to analyse the effects of depression as a potential confounder. The main hypothesis was that differences in cognitive performance between groups could perhaps be explained by depression for most cognitive domains but that some of them would also show a specific effect attributable to fibromyalgia. We were also interested in studying the possible effects of interaction between depression and fibromyalgia.

## Materials and methods

### Study design and setting

The study received the approval of the institutional ethics committee and the research was performed in accordance with the Helsinki Declaration. Written informed consent was obtained from all participants prior to their taking part in the study. We developed a matched case-control cross-sectional study including women from 30 to 55 years old who were divided into three groups: a group of patients who had been diagnosed with fibromyalgia (FM group, 110 patients), a group of patients with a depressive disorder (Depressive group, 33 patients), and a group of healthy subjects (Healthy group, 50 subjects). These were then matched by age (30–34, 35–39, 40–44, 45–49 and 50–55 years old) and years of schooling (8 years, 9–10 years, 11–12 years, 13–15 years and over 15 years of schooling). The recruitment was performed between August 2012 and November 2014. The entire protocol was administered by two experts in neuropsychology. The protocol was administered on 2 separate days for each participant, at the same time of day and with a maximum separation of 2 weeks between the first and second sessions. The tests were administered in the same order for all participants.

### Participants

The common inclusion criteria were women aged 30–55 years. Patients in the FM group were recruited at the Fibromyalgia Unit of the GSS-Hospital Universitari de Santa Maria, Lleida (Spain). The inclusion criteria for this group was to have been diagnosed with fibromyalgia by a rheumatologist specialised in this pathology and according to the 1990 ACR diagnosis criteria and to be on stable doses of medications at the time of the study. Exclusion criteria were: (a) history of neurological disorders or head trauma; (b) history of diagnosis of psychotic spectrum; (c) history of diagnosis of personality disorder; (d) history of nutritional or metabolic disease associated with cognitive dysfunction; (e) history of dependence on psychoactive substances; (f) evidence of a low estimated IQ, with a standard score of below 85 in the Vocabulary subtest of the Wechsler Adult Intelligence Scale 3^rd^ edition, WAIS-III [34]; (g) ongoing treatment with neuroleptic drugs; (h) cognitive impairment at the dementia level, with a score of less than 25 on the Mini Mental State Examination [35]; (h) diagnosis of other chronic autoimmune rheumatic diseases; and (i) lack of fluency in the Spanish language.

Patients in the Depressive group were recruited from the Mental Health Unit of the GSS-Hospital Universitari de Santa Maria, Lleida (Spain). The requirement for inclusion in this group was to have been diagnosed as suffering from depression by a psychiatrist from the Mental Health Unit, meeting the clinical criteria for Dysthymic Disorder, Major Depressive Disorder or Adjustment Disorder with Depressed Mood for the DSM-IV-TR, and having a score of over 13 on the Spanish adaptation of the Beck Depression Inventory-II (BDI-II) [36,37]. The exclusion criteria for the depressive group were not to present the specification of melancholic characteristics or atypical characteristics in their diagnosis of mood disorder. These patients were matched with the FM group by age, and education. In addition, they were matched with FM patients with BDI-II higher than 13 by the severity of their depressive symptoms measured by the BDI-II) considering three different levels of depression: low (scores between 14–19), moderate (scores between 20–28) and high (scores greater then 28).

Subjects from the Healthy group were selected from non-health care community settings. The inclusion criteria for this group were: not presenting any self-reported depressive symptoms or memory complaints and having a score of below 13 in the BDI-II and a score of over 12 on the Spanish adaptation of the General Health Questionnaire (GHQ-28). We used the revised score procedure (CGHQ) taking into account the chronicity of psychiatric symptoms [38,39]. This group was then matched with the FM group by age and education level, with a FM-Healthy ratio of 2:1.

The Depressive group and the Healthy group shared the same exclusion criteria as the FM group with the addition of the presence of any rheumatologic diagnosis, including Fibromyalgia.

### Cognitive assessment

For the cognitive assessment, we selected a battery of neuropsychological tests that have been related to the measurement of various components of attentional skills and executive functions. These cognitive tasks are listed and categorised in Table 1, described briefly below, and described in detail in S1 Appendix. Some tasks were administered by computer, using the Psychology Experiment Building Language (PEBL): license-free psychology software available from the program http://pebl.sf.net. Other tasks were administered with auditory or paper stimuli and oral responses.

**Table 1:** Cognitive domains assessed and neuropsychological tasks.

Cognitive Domains	Neuropsychologi-cal Tests	Outcomes	Method of Administration
Short term verbal memory	Digit Span Forward [34]	Digit span	Auditory / Oral
Selective Attention	d2 Test of Attention [40]	d2-TR	Visual / Manual (Paper)
		d2-TA	
		d2-TOT	
		d2-Con	
Sustained Attention and Impulsivity	CPT [41]	CPT Omission errors	Visual / Keyboard (PEBL)
		CPT Comission errors	
Processing Speed and Inhibition	Stroop Test [42]	Stroop Reading Words	Visual / Oral
		Stroop Color Naming	
		Stroop Word-Color	
		Stroop Interference Index	
Set-Shifting	Trail Making Test [43]	TMTBA	Visual / Manual (Paper)
Working Memory	N-back [44]	1-back, correct respons.	Visual / Keyboard (PEBL)
		2-back, correct respons.	
		3-back, correct respons.	
	PASAT [45]	PASAT 3.0	Auditory / Oral
		PASAT 2.0	
Response Inhibition	Go-NoGo Task [46]	Go errors	Visual / Keyboard (PEBL)
		No-Go errors	
		Reaction Time Go resp.	
		Reaction Time NoGo resp.	
Abstraction	BCST [47]	BCST, categories	Visual / Keyboard (PEBL)
Flexibility		BCST, perseverative errors	

Notes: CPT, Continuous Performance Test; PASAT, Paced Auditory Serial Addition Test; BCST. Berg Card Sorting Test; PEBL, Psychology Experiment Building Language; d2-TR, total responses; d2-TA, correct answers; d2-TOT, total test effectiveness; d2 Con, Concentration index (see S1 Appendix for a more detailed description)

#### Digit Span Forward [34]

This measure was used to measure short-term verbal memory. It involved the immediate recall of increasingly longer strings of digits that were read to the subjects.

#### D2 Test of Attention [40]

This test of selective attention includes 14 lines with the letters “d” and “p” accompanied by a different number of strokes. Participants were requested to cross out successive “d”s accompanied by two strokes on each line and to do this as quickly as possible and within a period of 20 seconds.

#### Continuous Performance Test [41]

In this task, subjects had to respond by pressing a button to target the letters presented on a computer screen, except when the letter “X” appeared. In the present study we used the number of commission errors (when a response was given after a letter “X” appeared on the screen) as a measure of impulsivity. The number of omission errors (when the participant failed to respond to the target stimulus) was taken as a measure of inattention.

#### Stroop Test [48]

This test uses a Word Card with 100 colour words (red, blue and green) printed in black ink, a Colour Card with 100 Xs printed in blue, red or green ink, and a Colour–Word Card with 100 names of colours printed in incongruent colours. The Word Card test provides a measure of automatic processing speed; the Colour Card test measures controlled processing speed; and the Colour-Word Card test examines cognitive flexibility. In addition, and Interference Index was also calculated by subtracting the number acquired in the Colour–Word Card from the number recorded in the Colour Card; thix index was used as a measure of the inhibition ability.

#### Trail Making Test [49]

This was a set of visual searches and sequencing tasks involving motor speed, attention and the ability to alternate between categories (set-shifting). In TMT-A, subjects are asked to connect numbers consecutively (e.g., 1-2-3), whereas on TMT-B, they must alternate between consecutive numbers and letters (e.g., 1-A-2-B). Scores are based on the length of time taken to complete each part of the test. We used the TMTBA: a composite score obtained by subtracting the time taken to complete part A from time taken to complete part B.

#### N-back Paradigm [44]

This task, which assesses working memory, was performed by computer. Subjects are required to monitor a continuous sequence of digits and to respond whenever the stimulus presented is the same as the one presented in *n* previous trials, where *n* is 1 (1-back condition), 2 (2-back condition) and 3 (3-back condition). The total scores ranged from 0 to 18, with higher scores indicating the best performance.

#### Paced Auditory Serial Addition Task [45]

Subjects add up consecutive numbers ranging from 1–9, which are presented by auditory tape, and respond orally with their sum. As each digit is presented, patients sum that number to the digit that was presented before it. There are two trials, with different presentation rates: in trial 1, the numbers are presented at three second intervals (PASAT 3.0) and in trial 2 at two second intervals (PASAT 2.0). The score for each trial was the number of correct responses for the 60 different combinations presented.

#### Go-NoGo Task [46]

This is a task that requires a response inhibition, in which participants are required to watch a sequence of the letters “P” and “R” presented on a computer screen. There were two conditions: under the first (Go condition), the participants were asked to press a button in response to the letter “P” and to withhold their response to the letter “R”; under the second (NoGo condition), they were asked to respond to the letter R and to withhold their response to the letter P.

#### Berg Card Sorting Test-64 [47]

The Berg Card Sorting Task (BCST) is a computerised version of the Wisconsin Card Sorting Test for assessing the ability of the individual to change their cognitive strategies in response to changing environmental contingencies. Participants were asked to sort a series of cards, based on simple stimuli, which were characterised by three relevant categories (colour, form, and number) with respect to four reference cards. The rules for correctly sorting the cards were modified during the course of the test. Participants had to complete a total of 48 different trials.

#### WAIS-III-R Vocabulary subtest

We used the vocabulary subtest from the Weschler-III scale [34] and scaled the score to provide a measure of premorbid intelligence. The participants provided definitions of words presented in an order of increasing difficulty.

### Assessment of self-reported depressive symptoms

We administered the Spanish version of the Beck Depression Inventory, BDI-II [50] to quantify the severity of depressive symptoms. This contains 21 items to assess the emotional, behavioural and somatic symptoms associated with depression. The range of scores is from 0 to 63 points. A total score of 13 or less is considered to indicate an absence of depression, 14–19 indicates mild depression, 20–28 indicates moderate depression and a score of 29 or more indicates severe depression.

### Assessment of anxiety symptoms and sleep quality

The Spanish version of the State-Trait Anxiety Inventory, STAI [51] was used to evaluate the intensity of anxiety. The test includes two subscales, each composed of 20 items: the State subscale measures anxiety related to a specific situation or period (at the moment of questionnaire completion), while the Trait subscale measures relatively stable anxiety. Total scores range from 20 to 80 for each subscale, with higher scores indicating greater anxiety. We chose the 75th percentile to normative data as our cut off for clinically significant levels of anxiety; this corresponded to a raw score of 31 for STAI-State and a raw score of 32 for STAI-Trait [52].

The assessment of sleep quality was conducted using the Pittsburgh Sleep Quality Index questionnaire (PSQI) adapted for Spain [53]. It differentiates between “poor” and “good” sleep, measuring seven different areas: subjective sleep quality, sleep latency, sleep duration, habitual sleep efficiency, sleep disturbances, use of sleeping medication, and daytime dysfunction during the previous month. The range of scores is from 0 to 21, where higher scores indicate lower sleep quality and a global sum of “5” or more indicates “poor” sleep quality.

### Statistical analysis

The mean and standard deviations were calculated to describe the demographic and clinical characteristics of the sample. A one-way ANOVA was used to assess differences between groups. When normality could not be assumed (a Kolmogorov-Smirnov test was used to asses this) a Kruskal-Wallis test was performed. The Bonferroni correction was also applied to adjust for multiple comparisons. To compare the cognitive performance for each sample group, the ANCOVA test was applied, adjusted for depression severity (BDI-II), anxiety (STAI-S, STAI-R) and sleep dysfunction (PSQI). A secondary analysis, using linear models, was performed to evaluate the joint effects of fibromyalgia and depression, as binary covariates, on cognitive measures. The models considered the main effects of depression and fibromyalgia and their potential interaction. As a result, four groups of patients were implicitly considered: subjects with both depressive symptoms and fibromyalgia; subjects with depressive symptoms but without fibromyalgia; subjects without depressive symptoms but with fibromyalgia; and subjects without either depressive symptoms or fibromyalgia. F-tests were used to compare the models for each outcome and to subsequently determine the model that showed the best performance. In this way, we were able to explore whether the effects observed were attributable to depression, to fibromyalgia, to their independent combination or to their interaction. Analyses were made using the SPSS 22 program for Windows (SPSS Inc.) and the R package and setting the significance level to 5% (α = 0.05) for all of the analyses.

## Results

### Recruitment results and characteristics of the sample

**Fig 1:**
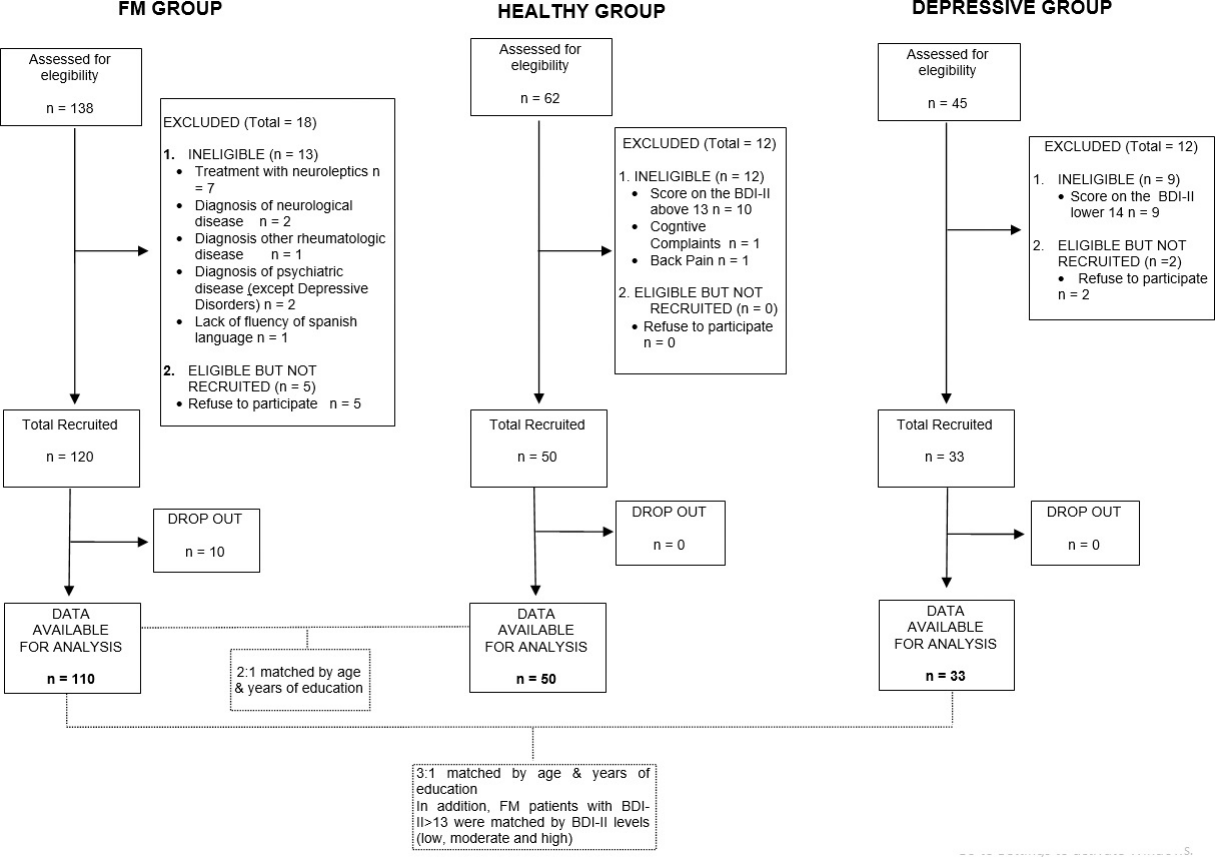
Flow chart of recruitment for the study.

Details of the recruitment results are shown in Fig 1. In the FM group, a total of 138 patients were initially selected, of which 28 were subsequently excluded: 13 were ineligible because they did not meet the entry criteria, while 5 decided not to participate due to a lack of time and interest. A further 10 subjects left the study before completing the whole assessment protocol due to bad health. As a result, this group was finally formed by a total of 110 subjects. In the Depressive group, 45 subjects were screened, of whom 9 were ineligible because they did not meet the entry criteria and 2 refused to participate; this group was therefore reduced to a total of 33 subjects. In the Healthy control group, 62 subjects were screened, of whom 12 were ineligible because they did not meet the entry criteria; this group therefore had a total of 50 subjects.

### Demographic and clinical variables

The demographic and clinical characteristics of the groups are presented in Table 2. The groups did not differ in either age or education. However, significant overall group differences were found for Vocabulary *(p* = 0.005), BDI-II *(p<0*.*001*), STAI-State *(p<0*.*001*), STAI-Trait *(p<0*.*001*) and PSQI scores *(p<0*.*001*). In the FM group, 86% of subjects scored within the range of depressive symptoms (whether mild, moderate or severe). Regarding anxiety scores, 68% of the patients in the FM Group scored within the range associated with significant levels of anxiety, compared with 78% of patients in the Depressive Group and 4% in the Healthy Group. Further, 99% of the patients in the FM Group were classified as a poor sleepers, whereas 94% in the Depressive Group and 40% of the patients in the Healthy Group. Follow-up analyses revealed that the FM Group and the Depressive Group did not differ in their Vocabulary, BDI-II or STAI scores (p>0.05), whereas the FM Group showed higher scores in PSQI than the Depressive Group *(p = 0*.*005)*. Both FM and the Depressive group presented higher scores in BDI-II, STAI and PSIQI scores than the Healthy group and lower scores in the Vocabulary subtest.

**Table 2:** Demographic and clinical characteristics for samples groups.

	Fibromyalgia Group (N = 110)	Depressive Group (N = 33)	Healthy Group (N = 50)	FM–Depress. *p-value* *^a^*	FM–Healthy *p-value* *^a^*
Age (years)	45.4 (5.6)	47.7 (5.6)	46.5 (6.5)	0.1	0.8
Education (years)	10.5 (2.8)	11.1 (2.9)	11.2 (3.0)	0.7	0.4
Vocabulary	38.5 (7.8)	41.4 (6.8)	42.4 (7.2)	0.2	0.007
BDI-II	26.5 (12.4)	25.7 (9.9)	3.6 (3.7)	1.0	<0.001
STAI-State	37.0 (11.0)	37.8 (9.0)	16.0 (9.5)	1.0	<0.001
STAI-Trait	35.8 (10.9)	34.6 (10.3)	12.7 (7.9)	1.0	<0.001
PSQI	11.9 (3.3)	9.9 (3.1)	4.7 (3.0)	0.005	<0.001

BDI-II. Beck Depression Inventory-second edition; STAI. State-Trait Anxiety Inventory; PSQI. Pittsburgh Sleep Quality Index

^a^ ANOVA test with Bonferroni correction

There were 89 patients in the FM group and 39 in the Depression group who were receiving pharmacological treatment (81% and 91% respectively of the total). None of the participants in the control group were taking any medications. The drugs that they were taking corresponded to the followings: analgesics and non-steroidal anti-inflammatory drugs (70*%* in the FM group and 10% in the Depression group); antidepressants (49*%* in the FM group and 91% in the Depression group); benzodiazepines (46% in the FM group and 67% in the Depression group); and opiates (20*%* in the FM group and 0,03% in the Depression group). All of the participants who were taking medication were on stable doses of treatment.

### Neuropsychological performance by groups

Table 3 shows results comparing performance in neuropsychological tests for the three groups, adjusting for depression (BDI-II), anxiety (STAI-S, STAI-T) and sleep quality (PSQI). The groups did not significantly differ in the Digit Span, CPT errors, Stroop Reading Words and Color Naming, TMTBA, all of n-back tasks, PASAT 3.0 and 2.0, Go-NoGo errors, Go-NoGO time responses and BCST category tests. In contrast, differences between groups were found in all of the d2 tasks (p<0.001), Stroop Word Color (p = 0.008), Stroop Interference Index (p = 0.03) and BCST perseverative errors (p = 0.006). Follow-up analyses showed that the FM group performed significantly worse than the Healthy group on the Stroop Word-Color and the Interference Index tests (p = 0.02 and p = 0.03 respectively). Comparisons of the FM and Depressive groups showed that the FM group performed significantly worse on the BCST perseverative errors test (p = 0.004), but significantly better on all the D2 tasks (p<0.001).

**Table 3:** Comparison of performance on the cognitive tests for each group and p-values for multiple comparisons adjusted for depression, anxiety, sleep quality and Vocabulary.

	Fibromyalgia Group (N = 110)	Depressive Group (N = 33)	Healthy Group (N = 50)	^a^ FM–Depress	^a^ FM–Healthy
Digit span	4.8 (0.9)	4.8 (0.9)	5.4 (1.0)	1.0	1.0
d2-TR	435.6 (90.0)	355.4 (80.3)	477.5 (87.3)	<0.001	0.6
d2-TA	165.9 (41.0)	134.1 (43.3)	185.4 (39.1)	<0.001	0.6
d2-TOT	415.2 (87.3)	335.4 (86.3)	446.2(106.3)	<0.001	0.3
d2-Con	163.8 (41.5)	131.2 (43.8)	182.6 (40.0)	<0.001	0.5
CPT Omission errors	4.0 (8.3)	2.2 (2.4)	1.0 (1.7)	1.0	1.0
CPT Commission errors	3.5 (1.8)	4.1 (2.3)	3.1 (2.3)	0.3	0.7
Stroop Reading Words	97.8 (15.3)	92.1 (16.9)	109.3 (15.2)	0.1	1.0
Stroop Color Naming	65.3 (10.0)	62.1 (12.4)	75.2 (10.6)	0.4	0.2
Stroop Word-Color	37.5 (8.8)	35.7 (9.9)	46.6 (8.2)	1.0	0.02
Stroop Interference Index	27.71 (8.90)	26.54 (8.04)	28.54 (8.08)	1.0	0.03
TMTBA	62.39 (39.0)	55.3 (38.4)	50.8 (57.7)	0.9	1.0
1-back. correct responses	16.7 (2.4)	17.2 (1.6)	17.5 (1.8)	1.0	1.0
2-back. correct responses	13.7 (4.0)	13.8 (4.1)	15.5 (2.6)	1.0	1.0
3-back. correct responses	11.1 (4.12)	11.2 (3.4)	13.3 (3.5)	1.0	0.5
PASAT 3.0	37.0 (15.4)	37.4 (12.6)	47.4 (8.7)	1.0	0.3
PASAT 2.0	23.8 (15.4)	26.0 (10.2)	34.5 (9.3)	1.0	0.6
Go errors	6.05 (4.7)	7.5 (5.9)	4.4 (3.4)	0.2	0.5
No-Go errors	1.3 (1.8)	1.4 (1.8)	0.7 (1.0)	1.0	0.3
Reaction Time Go responses	557.3 (87.0)	549.1 (84.0)	521.5 (47.7)	1.0	1.0
Reaction Time NoGo responses	621.3 (95.0)	610.9 (83.6)	590.1 (64.0)	1.0	1.0
BCST. categories	2.7 (1.3)	2.9 (1.3)	2.7 (1.2)	1.0	0.02
BCST. perseverative errors	13.3 (5.9)	9.6 (4.6)	11.4 (5.1)	0.004	1.0

Notes: CPT, Continuous Performance Test; PASAT, Paced Auditory Serial Addition Test; BCST. Berg Card Sorting Test; d2-TR, total responses; d2-TA, correct answers; d2-TOT, total test effectiveness; d2 Con, Concentration index

^a^ ANCOVA test adjusted for BDI-II, STAI-S, STAI-T and PSQI scores applying Bonferroni correction

Values are raw scores

### Associations with fibromyalgia, depression and interactions

Given our interest in deciphering whether or not the outcomes could be exclusively attributed to depressive symptoms, to fibromyalgia or to a combination of the two factors, we performed a secondary analysis. This consisted of separately studying the cognitive performance of patients with and without fibromyalgia divided into depressed and non-depressed groups. To do this, the participants were initially separated into 2 main groups: a group of patients that showed depressive symptoms and another group without depressive symptoms. The group with depressive symptoms was composed of subjects from the Depressive Group and of subjects from the Fibromyalgia Group with scores higher than 13 in the BDI-II. The group with non-depressive symptoms was composed of subjects from the Healthy Group and of subjects from the Fibromyalgia Group with scores equal to or lower than 13 in the BDI-II score. We then re-divided each of these groups according to whether the patients had been diagnosed with fibromyalgia or not. This gave us the following 4 subgroups: subjects with depressive symptoms and fibromyalgia; subjects with depressive symptoms without fibromyalgia; subjects without depressive symptoms and fibromyalgia; and subjects without depressive symptoms or fibromyalgia. (Table 4). Then, we performed a regression analysis considering the main effects and their potential interaction. First, results showed that some variables were not associated with either fibromyalgia or depression, TMTBA, 1-back, Go errors and reaction Time Go (F-test p-values >0.05 all). Second, some variables were only associated with depression, Digit Span, CPT Omission and Commission errors, 2-back, 3-back, PASAT 3.0, PASAT 2.0, NoGo errors and Reaction Time NoGo responses (F-test p-values 0.0005, 0.01, 0.08, 0.01, 0.001, 0.00004, 0.00002, 0.02 and 0.02, respectively). Third, a number of variables were associated with both fibromyalgia and depression, although without interaction, i.e. with and independent effect, d2-TA, d2-TOT and d2-Con (F-test p-values 0.003, 0.0004 and 0.002 respectively). Finally, an interaction between depression and fibromyalgia was detected for the d2-TR, Stroop Reading Words, Stroop Color Naming, Stroop Word-Color, Stroop Interference Index, BCST categories and BCST perseverative errors tests (F-test p-values 0.03, 0.003, 0.001, 0.004, 0.02, 0.03, 0.01 respectively). Detailed description of Table 4 including the beta values is presented in S1 Table.

**Table 4:** Mean and standard deviations for cognitive tests in patients with and without fibromyalgia separately in subjects with and without depressive symptoms.

*Not associated*	WITH DEPRESSIVE SYMPTOMS			WITHOUT DEPRESSIVE SYMPTOMS			A	B	C
	FM n = 96	No-FM n = 33	p-value^a^	FM n = 14	Non-FM n = 50	p-value^a^	p-value^b^		
TMTBA	62.49 (38.5)	55.30(38.4)	0.29	61.69 (44.1)	50.80 (57.70)	0.21	0.28	0.28	0.82
1-back. correct responses	16.73 (2.4)	17.18 (1.6)	0.28	16.28 (2.5)	17.48 (1.8)	0.02	0.26	0.06	0.34
Go errors	6.23 (4.8)	7.48 (5.90)	0.41	4.77 (3.4)	4.39 (3.4)	0.59	0.15	0.12	0.06
Reaction Time Go responses	553.98 (82.3)	549.07(84.1)	0.76	581.05 (83.1)	521.56 (47.7)	0.01	0.23	0.18	0.23
CPT Commission errors	3.51 (1.8)	4.08 (2.3)	0.32	3.19 (1.8)	3.08 (2.3)	0.50	0.08	0.31	0.38
***Associated only with depression***									
Digit span	4.8 (0.9)	4.82 (0.9)	0.71	4.93 (0.8)	5.42 (1.0)	0.08	**<0.001**	0.3	0.16
CPT Omission errors	4.30 (8.8)	2.24 (2.4)	0.84	1.79 (2.6)	1.03 (1.7)	0.41	***0*.*01***	0.14	0.59
2-back. correct responses	13.67 (4.1)	13.82 (4.1)	0.84	14.07 (3.1)	15.48 (2.6)	0.13	***0*.*01***	0.39	0.35
3-back. correct responses	11.01 (4.2)	11.21 (3.4)	0.84	11.86 (3.7)	13.3 (3.6)	0.19	***0*.*001***	0.37	0.38
PASAT 3.0	36.63 (15.5)	37.39 (12.6)	0.92	39.21 (14.5)	47.42 (8.7)	0.08	**<0.001**	0.18	0.13
PASAT 2.0	23.21 (15.1)	25.97 (10.2)	0.54	27.5 (17.1)	34.47 (9.3)	0.14	**<0.001**	0.07	0.38
No-Go errors	1.40 (2.1)	1.39 (1.8)	0.99	0.77 (0.6)	0.72 (1.0)	0.31	***0*.*02***	0.95	0.94
Reaction Time NoGo respon	619.04 (96.8)	610.90 (83.6)	0.78	637.20 (80.1)	590.14 (63.8)	0.01	**0.02**	0.95	0.94
***Associated with depression and fibromyalgia***									
d2-TA	162.36 (40.9)	134.15 (43.3)	<0.001	189.5 (39.4)	185.44 (39.1)	0.83	<0.001	***0*.*003***	0.1
d2-TOT	407.83 (86.8)	335.36 (86.3)	<0.001	463.28 (77.5)	446.22 (106.3)	0.84	<0.001	**<0.001**	0.1
d2-Con	160.02 (40.7)	131.18 (43.8)	0.001	188.35 (39.3)	182.56 (40.0)	0.74	<0.001	***0*.*002***	0.12
**Depression interacting with fibromyalgia**									
d2-TR	428.82 (89.8)	355.39 (80.3)	<0.001	480.21 (78.8)	477.46 (87.3)	0.90	<0.001	0.0005	***0*.*03***
Stroop Reading Words	97.83 (15.1)	92.15 (16.9)	0.23	97.92 (17.2)	109.28 (15.2)	0.055	<0.001	0.87	***0*.*003***
Stroop Color Naming	65.23 (10.1)	62.21 (12.4)	0.22	65.5 (9.6)	75.16 (10.6)	0.003	<0.001	0.62	***0*.*001***
Stroop Word-Color	39.00 (5.6)	37.03 (6.9)	0.35	39.13 (5.7)	44.42 (5.9)	0.005	<0.001	0.42	***0*.*004***
Stroop Interference Index	27.86 (9.0)	26.55 (8.0)	0.18	26.64 (8.6)	28.54 (8.1)	0.005	<0.001	0.46	***0*.*02***
BCST categories	2.62 (1.2)	2.97 (1.3)	0.17	3.36 (1.2)	2.69 (1.2)	0.09	0.54	0.89	***0*.*03***
BCST perseverative errors	13.74 (5.9)	9.61 (4.6)	<0.001	10.45 (5.1)	11.46 (5.1)	0.47	0.12	0.007	***0*.*01***

^a^ p-values from a t-test comparing FM and non-FM group

^b^p-values from F-test comparing (A) the null linear regression model and the linear regression model with the main effect of depression, (B) the linear regression models with the main effect of depression and with the main effects of depression and fibromyalgia; and (C) the linear regression models with the main effects of depression and fibromyalgia and their interaction, are also shown

Values are raw scores

**Fig 2:**
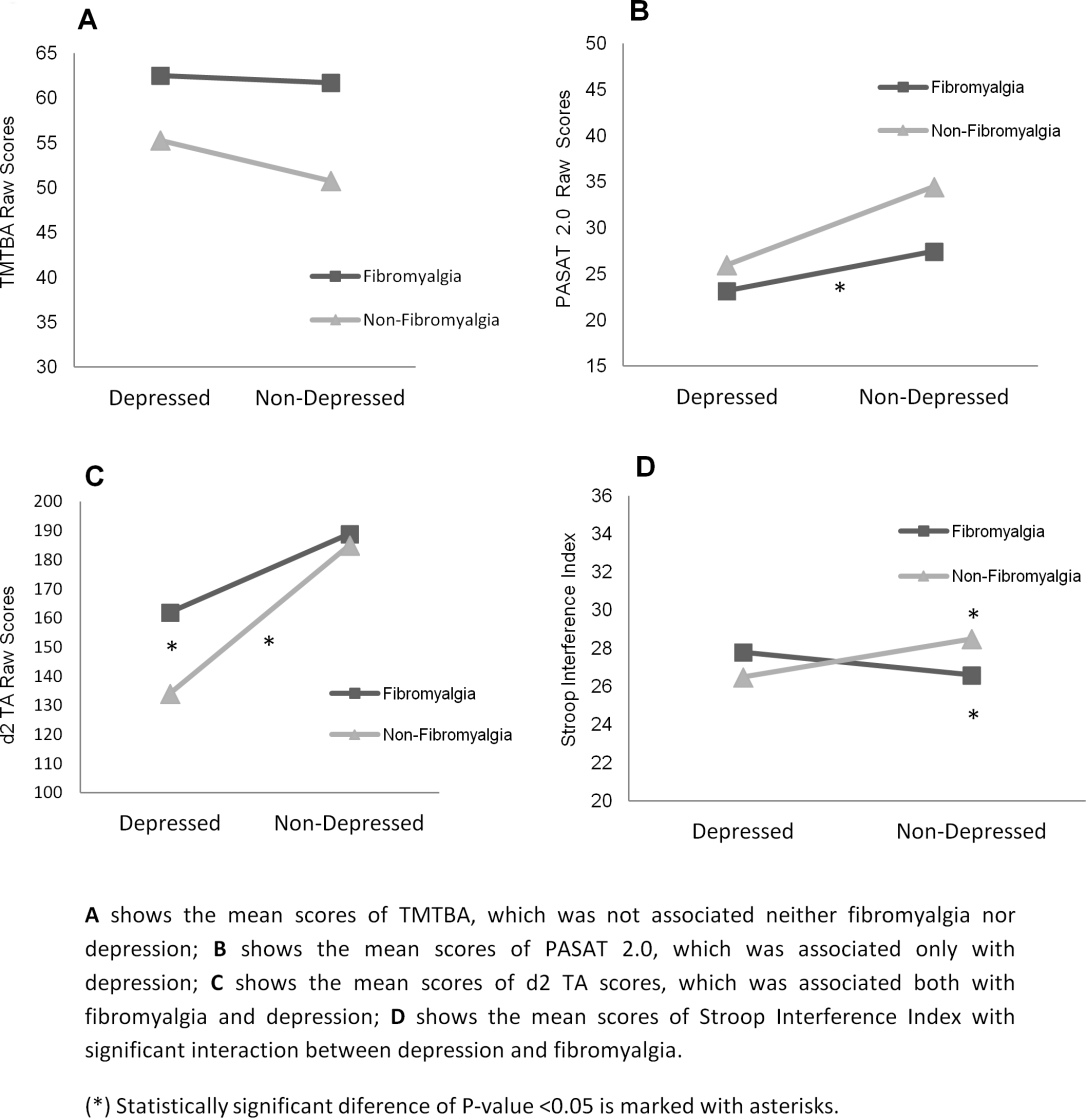
Mean comparisons in cognitive tests, separating patients with and without fibromyalgia and those who were depressed and non-depressed. These figures show the 4 different scenarios: A) no effect; B) an effect associated with depression; C) an effect associated with both depression and fibromyalgia; D) an effect associated with the interaction between the two.

Fig 2 allows us to see the performance in cognitive tests distinguishing between patients with and without fibromyalgia and who were depressed and non-depressed. In this way, we were able to ascertain that: neither fibromyalgia nor depression were associated with TMTBA performance (A); depression was the only factor associated with performance in the PASAT 2.0 (B); both fibromyalgia and depression were associated with performance in d2TA (C); and, depression and fibromyalgia showed interaction in the Stroop Interference Index, indicating that the effect of fibromyalgia in this measurement depended on whether the subject showed signs of depression or not (D).

## Discussion

In this study, we provide new evidence to further our understanding of the effects of FM on cognitive performance. We have done this separating the attributable effects of potential confounding variables and, in particular, the effects explained by depressive symptoms, and by also taking into account the influence of anxiety and sleep dysfunction.

First, and as expected, a very large discrepancy was found between the FM and Healthy groups with respect to measurements relating to depression, anxiety and sleep quality. The FM group showed high levels of depression, anxiety and sleep dysfunction: 86% of subjects scored within the range of depressive symptoms, 68% within the range associated with significant levels of anxiety and 99% showed poor sleep quality. These results agree with those of the vast majority of studies undertaken with FM patients: exhibiting a high presence of anxiety, depressive and sleep disorders in this population [18,54–56]. In contrast, the FM and Depressive groups did not differ in their depressive and anxious symptoms, although the FM group exhibited worse sleep quality than the Depressive group. This finding was in line with Choy [57], who suggested that sleep dysfunction might be pathogenic in fibromyalgia. This is also important because it has been identified as one of the main domains interfering with patient quality of life [58].

With regard to the specific objectives of the study in terms of cognitive performance, the first finding was that the differences initially found between the FM and the Healthy groups in the majority of the cognitive measurements subsequently disappeared when we adjusted for depression, anxiety and sleep quality, leaving only differences in the Stroop Interference task. This lack of difference in most of the cognitive tests supports our main hypothesis that the differences in cognitive performance between groups could mainly be explained by depression for most cognitive domains. Moreover, this is also in line with earlier studies that showed no significant differences in the cognitive performance of patients with fibromyalgia when depression components were controlled [10,22,59]. The results presented by Sletvold [11] and Landrø [12], who also studied cognitive performance in FM patients, comparing their performance with those of a healthy control group and a group of patients with Major Depression, ran along the same lines. They found that both patients with FM and depression shared a nonspecific deficit in their capacity to process information and long-term recall memory. Even so, other studies involving FM patients failed to find any relationship between the intensity of the depressive symptoms and cognitive performance [4,23,25,33,60]. It is also worth mentioning the fact that there were significant methodological differences between these studies and our own, as many of the previous studies did not include patients with depression, whether because this had been an exclusion criterion or because the intensity of the depressive symptoms had been either null or mild over their whole sample. As a result, those samples were different from ours and it is not, therefore, strange to find different results. Our experience in working with patients with FM leads us to believe that the high proportion of depressive symptoms found in the patients who participated in our study should not be regarded as unusual.

The association identified in this study in the Interference task of the Stroop Test had also been previously demonstrated in other studies involving patients with FM. Martinsen et al [61] found pronounced differences between FM patients compared to healthy controls in the incongruent condition with respect to the congruent condition of this same task. This was also associated with a reduced activation of the caudate nucleus, lingual gyrus, temporal areas and the hippocampus in FM patients. Similarly, Mercado et al [62] found a significant emotional Stroop interference effect in FM patients when recording their brain activity with event-related potentials. The results of this study revealed that the cognitive inhibition associated with a very automatic response elicited greater prefrontal neural activity when symptom-related stimuli were processed. These results were only observed for the FM group which led the authors to suggest a specific difficulty in cognitive inhibition in fibromyalgia patients under conditions linked with their disease. These results highlight the problems identified in this population with respect to the ability to detect conflicts and automatically inhibit unwanted irrelevant responses. This is a capacity that allows individuals to regulate information processing in order to deal with a concurrent task and constitutes a central component of executive control and one that is critical for inhibiting response tendencies [63].

The second important finding of this study concerns the comparison between the FM and Depressive groups with respect to cognitive performance. Both groups showed similar levels of performance in most of the cognitive tests, with the exception of measures of selective attention and flexibility. The Depressive group showed the worst performance in all of the tasks in the d2 Test of Attention. Several previous studies have identified cognitive dysfunction in depressed patients, especially affecting the domains of attention, memory, psychomotor speed, processing speed and executive function [19–21]. The FM group also showed a greater number of perseverative errors in the BCST test than the Depressive group, suggesting that this could be a specific dysfunction related to fibromyalgia. The results from Walteros et al. [59] were similar; they found that FM patients displayed more perseveration errors in CALT, an experimental learning task designed to assess the acquisition of arbitrary associations between targets and colours [64]. This impairment of cognitive flexibility in fibromyalgia patients would also be compatible with passive and maladaptive coping responses that have been described in individuals with pain-related conditions [65].

The secondary analysis performed in this study to separately evaluate the effects of depression and fibromyalgia on cognitive tests allowed us to refine our main hypothesis that differences in cognitive performance between groups could be explained by depression for most cognitive domains but that some would exhibit a specific effect attributable to fibromyalgia. Based on the results of this secondary study, and taking into account the high comorbidity between fibromyalgia and depression, FM patients might be expected to perform more poorly on tasks involving short term memory, working memory, selective attention, flexibility, processing speed, inhibition and flexibility. Some of this impaired performance seems to be related to depression (short term memory, working memory and inattention), other parts to the additive effect of FM and depression (selective attention) and the rest to the effects of interaction between FM and depression (processing speed, inhibition and flexibility). Some of this impaired performance would be related to depression (short term memory, working memory and inattention), other to the additive effect of FM and depression (selective attention) and the rest to the interactive effect of FM and depression (processing speed, inhibition and flexibility). Special attention should be given to these significant interactions and their interpretation: the effect of depression in these cognitive tasks is different depending on whether or not the person has FM. The final result did not, therefore, correspond to the sum of these 2 factors, but had an effect of its own. In d2TR, a selective attention task that also measured processing speed, we found that patients with FM and depression performed better than those without FM and depression. This suggests that FM could have acted as a protector against the clearly negative effects of depression as far as processing speed and selective attention were concerned. It would thus have minimised the effects that depression could have had on its own. This was not, however, maintained in the rest of tasks that also measured this function, such as the Stroop Reading Words and Stroop Word Colors tests. In these tasks, the main differences were found in the non-Depression group. In them, the patients with FM showed the worst levels of performance. However, this poor result was not maintained in the group of people with depression; they did not present any significant differences in performance for this task. This may suggest that when depression is present, the effects of FM are matched by those of depression, but when there is no depression, the deleterious effects of FM are clearly visible. Similar conclusions were drawn in relation to inhibition as measured by the Stroop Interference Index. Finally, In the BCST perseverative errors task, the patients who showed the worst performance (those who produced the most perseverative errors) were the ones with both FM and depression. In the group without depression, on the other hand, there were no observable differences in the number of errors, whether the patients had FM or not. Although the reasons for these findings are likely to require further analysis, all of the interactions observed confirmed that the effects of the two key factors: depression and FM, should not be minimized. The possible effects of interactions between them should therefore be considered. What is more, these interactions could explain some of the discordances found between the results of different studies undertaken with this population, particularly considering that interactions between different variables have hardly ever been analyzed. This factor may also help to explain the high level of heterogeneity identified in these patients. To the best of our knowledge, this is a novel finding, although further studies will be required to validate these results. No differences were identified in the performance of the participants with respect to the cognitive load of the tests administered. This contrasted with the findings from another recent work carried out by the same authors [66].

One of the strengths of our study was that it included a reasonable range of neuropsychological tasks covering the main attentional and executive areas; another was the sample size, which was larger than in previous studies. Moreover, the design of this study allowed us to separate the effects of potential confounder variables from FM in relation to cognitive performance and to study interactions between different variables.

However, our study also presents some methodological limitations. First, there may have been a degree of selection bias, because it was not possible to randomize the selection of participants; instead, it was the subjects themselves who decided whether or not to participate in the study. Even so, it is worth mentioning that refusal to participate in the study was minimal, in both the patient and control groups, as was the loss of cases during the assessment. Further, the present study only recruited women; this allowed us to provide greater homogeneity in the sample, but future studies should be replicated including men with fibromyalgia. Second, this is a case-control study and, therefore, only statistical association can be studied. No causality can be attributed to the explanatory variables (mainly fibromyalgia and depression) on the responses. Third, it is also important to remark that when we designed this study, we were aware of the unfeasibility of including the same number of controls than cases given that fibromyalgia patients exhibit a lower educational level than the general population. Given that the performance on cognitive tasks is related to the educational level, we were interested to focus on recruiting the largest number of cases, taking into account our actual possibilities, and matching pairs of them with one single control. However the statistical results obtained with the proposed design shows that we achieved enough power to solve our hypothesis. Furthermore, the matching ratio of cases with patients with depression was 3:1, given once again the unfeasibility of a 1:1 ratio which could have caused an increase of the type II error, with a consequent loss of statistical power. That said, the present study may well reflect the current reality of FM patients, and the fact that the control group was enriched with patients with depression allowed us to assess the effects of FM itself, having previously adjusted for depression. Larger observational studies including non-depressed FM patients should also be undertaken. In our study we limited our work to evaluating the presence or absence of a potential interaction between fibromyalgia and depression in the responses. In this respect, beta values and F statistics could also have been presented in Table 4, in addition to p-values, for interpretation purposes. We have intentionally omitted this information for the sake of simplicity; we believe that this deeper level of analysis could be reserved for future work, which would ideally include an increased sample size and perhaps also the fitting of non-linear models. Finally, we must also discuss the possible limitations associated with a lack of control over medication that the patients were taking, having excluded patients who were taking treatment with neuroleptics but allowing treatment with anxiolytic agents and antidepressants. It is important to note, however, that treatment with anxiolytics was at low doses for all patients. Further, the present study has specifically focused on analyzing the effect of depression as a potential confounder in the cognitive performance of patients with fibromyalgia. However, future studies should also include an in depth analysis of the role of anxiety as this is a symptom with a high prevalence in this disease and one that also has significant repercussions on cognitive functions.

In summary, these results lead us to conclude that fibromyalgia patients show characteristic cognitive dysfunction in their attentional and executive domains. While many of these can be explained by the effects of depressive symptoms, some others seem to also depend on the effects of fibromyalgia. More specifically, we found that short-term auditory memory, working memory and inattention scores were all associated with depression alone, whereas selective attention was associated with both depression and fibromyalgia, and processing speed, cognitive flexibility and inhibitory control showed significant interactions between depression and fibromyalgia.

From a clinical practice perspective, we must consider emotional symptoms, such as depression and anxiety, to be an essential part of the cognitive performance of patients with fibromyalgia; it therefore makes no sense to analyze them separately. All these results suggest that improving emotional symptoms in this population would reduce the impairment of attentional processes even though impairment of cognitive flexibility and inhibition would still remain. Our findings also highlight the importance of clinicians understanding this pattern of cognitive dysfunction in order to provide a specific approach that could be used to address both the emotional and cognitive domains and thus help to improve the cognitive performance of those suffering from fibromyalgia.

## Supporting information

S1 Appendix Description of the neuropsychological tests used in the study. (DOCX)

S1 Table Estimated parameters (beta) and standard deviations (SE) for cognitive tests in patients with and without fibromyalgia separately in subjects with and without depressive symptoms. (DOCX)
