# Supplementary material for: The effect of depressive symptoms on cognition in patients with fibromyalgia
Source: PLoS One. 2018 Jul 5;13(7):e0200057. doi: 10.1371/journal.pone.0200057 (PMC6033429; doi:10.1371/journal.pone.0200057)
Supplement: S1 Appendix — (DOCX) [file pone.0200057.s001.docx]

**S1 Appendix. Description of the neuropsychological tests used in the study**

Digit Span Forward [1]

This task was used to measure the short-term verbal memory, and it involved the immediate recall forward of increasingly longer strings of digits that were read to the subjects. The score was the length of the longest list that a subject could recall.

D2 Test o f Attention [2]

This instrument is a time test of selective and sustained attention that includes 14 lines with “d” and “p” that are marked with different numbers of strokes. Participants were requested to cancel successive each d with two strokes in each line as quickly as possible and with the fewest number of mistakes. For analysis values we used the total number of finished items (TR score), the total number of correct answers (TA score), and two composite scores, TOT score, obtained from subtracting errors from the number of total answers, and CON score, obtained from subtracting commission errors from the number of total correct answers. TR score was used as a measure of processing speed and the other measures were used to evaluate the selective attention.

Continuous Performance Test [3]

Sustained attention and impulsivity was assessed with the Psychology Experiment Building Language (PEBL) Continuous Performance Task (CPT). In this task, subjects have to respond by pressing a button to target letters presented on the computer screen except when the letter “X” appeared. In the present study we used the number of commission errors (when a response is given after an “X” letter appears on the screen) as a measure of impulsivity and the number of omission errors (when the participant fails to respond to the target stimulus) as a measure of inattention.

Stroop Color and Word Test [4]

This test consists of three cards, the Word Card, with 100 color words (red, blue, green) printed in black ink; the Color Card, with 100 Xs printed in blue, red, or green ink; and the Color–Word Card, with 100 names of colors printed in an incongruent color. Participants are asked to read the words (Word Card) or to name the ink color (Color and Color-Word Card) as quickly as possible with a time limit of 45 seconds. The Word Card provides a measure of automatic processing speed, and the Color Card a measure of processing controlled speed. In addition, an interference index was calculated by subtracting the number acquired in the word-color incongruent subtask from the number recorded in the color subtask, as suggested by the author [4]. This score was used as a measure of the efficiency of inhibitory mechanisms [5].

Trail Making Test [6]

The Trail Making Test is a set of visual search and sequencing tasks involving motor speed, attention and the ability to alternate between categories (set-shifting). On TMT-A, subjects are asked to connect numbers consecutively (eg, 1-2-3), whereas on TMT-B, they alternated between consecutive numbers and letters (eg, 1-A-2-B). Scores are the amount of time taken to complete each part. We were interested specifically in evaluating the set-shifting, and we used a score based on the subtraction of time to complete part A from time to complete part B.

N-back Paradigm [7]

This task, which assesses working memory, was performed by computer. Subjects were required to monitor a continuous sequence of letters and to respond, pressing a key, whenever the stimulus that was presented was the same as the one presented *n* trials previously, where n was 1 (1-back), 2 (2-back) and 3 (3-back). Total scores ranged from 0 to 18, with higher scores indicating better performance. Outcomes were the total number of correct responses in the each condition.

Paced Auditory Serial Addition Task -PASAT- [8]

This is a test which assesses working memory, as well as sustained attention, auditory processing speed, calculation ability and stimulus competition filtering skill [8]. Subjects add consecutive numbers 1–9 presented by auditory tape and respond orally with a sum. As each digit is presented, patients sum that number with the digit that was presented before it. In this study we used the version included in the Brief Repeatable Battery of Neuropsychological Test for Multiple Sclerosis [9] and auditory stimuli were presented through computer. There are two trials, with different presentations rates: in trial 1 presentation rates are 3.0 seconds (PASAT 3.0) and in trial 2 are 2.0 seconds (PASAT 2.0). The score for each trial is the number of correct responses over 60 digits presented.

Go-NoGo Task [10]

The Go-Nogo task is a computerized test of response time and response inhibition where a motor response must either be executed or inhibited. Our version was programmed using the Psychology Experiment Building Language (PEBL) [11]. During this task, participants were required to watch a sequential presentation of letters “P” and “R” on a computer and respond to a target letter by pressing a key. It consisted in two conditions, in the first condition (Go condition), participants were asked to press a button in response to the letter “P” and withhold their response to the letter “R”, and in the second condition (NoGo condition) participants were asked to make a response to the letter R and withhold their response to the letter P. The scores used were the number of errors in Go and NoGo conditions (“Go errors” and “NoGo errors”). Go errors were considered as an indicator of inattention, and NoGo errors were considered as indicator of impulsivity [12]

Berg Card Sorting Test-64 [13]

The Berg Card Sorting Task (BCST) is a computerized version of the Wisconsin Card Sorting Test assessing the ability to change cognitive strategies in response to changing environmental contingencies. The task was implemented in PEBL [11]. Participants were asked to sort a series of cards that had simple stimuli characterized by three relevant categories (color, form, and number) to four reference cards. The rules for correctly sorting the cards were modified during the performance of the test. Participants must complete a total of 48 trials. We chose the total number of categories achieved as a measure of abstract reasoning, and the total number of perseverative errors as a measure of cognitive flexibility.

WAIS-III-R Vocabulary subtest

We used the vocabulary subtest of the Weschler-III scale [1], scaled score, as a measure of premorbid intelligence. Participants provided definitions of words presented in the order of increasing difficulty

**References**

1. Wechsler D. WAIS - III Escala de Inteligencia de Wechsler para Adultos. Madrid: TEA Ediciones; 1999.

2. Brickenkamp R, Zillmer E. D2. Test de atención. Adaptación española de Nicolás Seisdedos Cubero [D2 Test of Attention. Spanish adaptation by Nicolás Seisdedos Cubero]. Madrid: TEA Ediciones; 2004.

3. Conners CK, Epstein JN, Angold A, Klaric J. Continuous performance test performance in a normative epidemiological sample. Journal of abnormal child psychology [Internet]. 2003 Oct [cited 2014 Feb 12];31(5):555–62. Available from: http://www.ncbi.nlm.nih.gov/pubmed/14561062

4. Golden C. Stroop Color and Word Test: A Manual for Clinical and Experimental Uses. In Chicago, Illinois: . pp.: Skoelting; 1978. p. 1–32.

5. Dempster FN. The rise and fall of the inhibitory mechanism: Toward a unified theory of cognitive development and aging. Developmental Review [Internet]. 1992;12(1):45–75. Available from: http://www.sciencedirect.com/science/article/pii/027322979290003K\npapers3://publication/uuid/6BC6D23F-18C5-4937-8B16-7DD8F917B430

6. Reitan RM. Validity of Trail Making Test as an indicator of organic brain damage. Perceptual and motor skills. 1958;8:271–6.

7. Owen AM, McMillan KM, Laird AR, Bullmore E. N-back working memory paradigm: a meta-analysis of normative functional neuroimaging studies. Human brain mapping [Internet]. 2005 May [cited 2014 Jul 14];25(1):46–59. Available from: http://www.ncbi.nlm.nih.gov/pubmed/15846822

8. Gronwall DM. Paced auditory serial-addition task: a measure of recovery from concussion. Perceptual and motor skills [Internet]. 1977 Apr [cited 2014 Feb 17];44(2):367–73. Available from: http://www.ncbi.nlm.nih.gov/pubmed/866038

9. Rao SM, Leo GJ, Bernardin L, Unverzagt F. Cognitive dysfunction in multiple sclerosis. Frequency, patterns, and prediction. Neurology. 1991;41:685–91.

10. Bezdjian S, Baker LA, Lozano DI, Raine A. Assessing inattention and impulsivity in children during the Go/NoGo task. British Journal of Developmental Psychology [Internet]. 2009 Jun [cited 2014 Feb 12];27(2):365–83. Available from: http://www.pubmedcentral.nih.gov/articlerender.fcgi?artid=2757760&tool=pmcentrez&rendertype=abstract

11. Mueller ST, Piper BJ. The Psychology Experiment Building Language (PEBL) and PEBL Test Battery. Journal of neuroscience methods [Internet]. 2014 Jan 30 [cited 2014 Feb 3];222:250–9. Available from: http://www.ncbi.nlm.nih.gov/pubmed/24269254

12. Barkley RA. The ecological validity of laboratory and analogue assessment methods of ADHD symptoms. Journal of abnormal child psychology [Internet]. 1991 Apr [cited 2016 Nov 20];19(2):149–78. Available from: http://www.ncbi.nlm.nih.gov/pubmed/2056161

13. Berg EA. A simple objective technique for measuring flexibility in thinking. The Journal of general psychology [Internet]. 1948 Jul [cited 2014 Feb 4];39(1):15–22. Available from: http://www.ncbi.nlm.nih.gov/pubmed/18889466
