# Supplementary material for: The effect of depressive symptoms on cognition in patients with fibromyalgia
Source: PLoS One. 2018 Jul 5;13(7):e0200057. doi: 10.1371/journal.pone.0200057 (PMC6033429; doi:10.1371/journal.pone.0200057)
Supplement: S1 Table — (DOCX) [file pone.0200057.s002.docx]

S1 Table. Estimated parameters (beta) and standard deviations (SE) for cognitive tests in patients with and without fibromyalgia separately in subjects with and without depressive symptoms

|  | **Model with depression** | | **Model with depression and fibromyalgia** | | | | **Model with depression, fibromyalgia an their interaction** | | | | | |
| --- | --- | --- | --- | --- | --- | --- | --- | --- | --- | --- | --- | --- |
| Cognitive Tests | Depression  Beta (SE) | p-value | Fibromyalgia  Beta (SE) | p-value | Depression  Beta (SE) | p-value | Fibromyalgia  Beta (SE) | p-value | Depression  Beta (SE) | p-value | Interaction Fibromyalgia x Depression  Beta (SE) | p-value |
| Digit span | -0.53 (0.14) | 0.0003 | -0.15 (-0.15) | 0.32 | -0.45 (0.16) | 0.007 | -0.42 (-0.42) | 0.13 | -0.60 (0.21) | 0.005 | 0.39 (0.33) | 0.24 |
| d2-TR | -70.91(13.76) | <0.00001 | 51.25(51.25) | 0.0006 | -96.69(15.24) | <0.00001 | 7.54 (7.54) | 0.77 | -122.06 (19.39) | <0.00001 | 64.54 (30.93) | 0.04 |
| d2-TA | -32.78 (6.3) | <0.00001 | 20.85(20.85) | 0.002 | -43.27 (7.03) | <0.00001 | 7.03 (7.03) | 0.55 | -51.28 (9) | <0.00001 | 20.40 (14.36) | 0.16 |
| d2-TOT | -63.95(14.48) | 0.00002 | 55.32(55.32) | 0.0004 | -91.78(16.01) | <0.00001 | 22.45 (22.45) | 0.4 | -110.8 (20.49) | <0.00001 | 48.53 (32.68) | 0.14 |
| d2-Con | -32.85(6.41) | <0.00001 | 21.83(21.84) | 0.002 | -43.83 (7.14) | <0.00001 | 8.84 (8.84) | 0.46 | -51.38 (9.14) | <0.00001 | 19.19 (14.58) | 0.19 |
| CPT Commission errors | 0.52 (0.32) | 0.11 | -0.32 (-0.32) | 0.36 | 0.68 (0.37) | 0.06 | 0.25 (0.25) | 0.7 | 1.00 (0.47) | 0.03 | -0.83 (0.76) | 0.28 |
| CPT Omission errors | 2.59 (1.01) | 0.01 | 1.67 (1.67) | 0.13 | 1.73 (1.15) | 0.14 | 0.751 ( 0.75 ) | 0.7 | 1.20 (1.48) | 0.42 | 1.35 (2.37) | 0.57 |
| Stroop Reading Words | -10.76(2.42) | 0.00001 | 0.51 (0.51) | 0.85 | -11.02 (2.78) | 0.0001 | -10.01 (-10.01) | 0.03 | -17.13 (3.51) | <0.00001 | 15.48 (5.58) | 0.006 |
| Stroop Color Naming | -8.72 (1.66) | <0.00001 | -0.90 (-0.9) | 0.62 | -8.27 (1.9) | 0.00002 | -8.96 (-8.96) | 0.005 | -12.95 (2.39) | <0.00001 | 11.86 (3.8) | 0.002 |
| Stroop Word-Color | -7.85 (1.38) | <0.00001 | -1.38 (-1.38) | 0.36 | -7.14 (1.58) | 0.00001 | -7.95 (-7.95) | 0.003 | -10.95 (1.99) | <0.00001 | 9.67 (3.17) | 0.003 |
| Stroop Interference Index | -4.87 (0.92) | <0.00001 | -0.25 (-0.25) | 0.8 | -4.74 (1.06) | 0.00001 | -4.81 (-4.82) | 0.006 | -7.39 (1.33) | <0.00001 | 6.71 (2.11) | 0.002 |
| TMTBA | 6.56 (6.88) | 0.34 | 8.85 (8.86) | 0.24 | 1.99 (7.88) | 0.8 | 13.42 (13.42) | 0.32 | 4.51 (10.05) | 0.65 | -6.60 (16.27) | 0.69 |
| 1-back. correct responses | -0.32 (0.33) | 0.33 | -0.70 (-0.7) | 0.049 | 0.04 (0.37) | 0.92 | -1.28 (-1.28) | 0.04 | -0.30 (0.48) | 0.53 | 0.85 (0.76) | 0.27 |
| 2-back. correct responses | -1.39 (0.56) | 0.01 | -0.58 (-0.58) | 0.35 | -1.10 (0.65) | 0.09 | -1.55 (-1.55) | 0.16 | -1.67 (0.83) | 0.046 | 1.42 (1.32) | 0.28 |
| 3-back. correct responses | -1.70 (0.59) | 0.005 | -0.73 (-0.73) | 0.26 | -1.33 (0.68) | 0.0503 | -2.03 (-2.03) | 0.08 | -2.09 (0.87) | 0.02 | 1.92 (1.38) | 0.17 |
| PASAT 3.0 | -8.54 (2.08) | 0.00006 | -3.29 (-3.29) | 0.15 | -690 (2.36) | 0.004 | -8.69 (-8.69) | 0.03 | -10.03 (3.02) | 0.001 | 7.98 (4.81) | 0.1 |
| PASAT 2.0 | -8.50 (2.06) | 0.00006 | -4.39 (-4.39) | 0.049 | -6.32 (2.32) | 0.007 | -8.14 (-8.14) | 0.04 | -8.5 (2.98) | 0.005 | 5.53 (4.74) | 0.25 |
| Go errors | 2.11 (0.73) | 0.004 | -0.76 (-0.76) | 0.34 | 2.49 (0.83) | 0.003 | 0.323 ( 0.32 ) | 0.82 | 3.09 (1.06) | 0.004 | -1.56 (1.71) | 0.36 |
| No-Go errors | 0.69 (0.27) | 0.01 | 0.012 (0.01) | 0.97 | 0.68 (0.31) | 0.03 | -0.003 ( 0 ) | 1 | 0.68 (0.4) | 0.09 | 0.022 (0.64) | 0.97 |
| Reaction Time Go responses | 20.13(12.42) | 0.11 | 19.63(19.63) | 0.14 | 10.27(14.07) | 0.47 | 50.495 ( 50.49 ) | 0.04 | 27.51 (17.91) | 0.13 | -44.51 (28.78) | 0.12 |
| Reaction Time NoGo responses | 18.61 (13.6) | 0.17 | 18.37(18.37) | 0.21 | 9.26 (15.5) | 0.55 | 38.586 ( 38.59 ) | 0.14 | 20.76 (19.88) | 0.3 | -29.35 (31.76) | 0.36 |
| BCST. categories | -0.54 (1.03) | 0.6 | 1.75 (1.75) | 0.12 | -1.37 (1.16) | 0.24 | 4.579 ( 4.58 ) | 0.02 | 0.28 (1.47) | 0.85 | -4.20 (2.35) | 0.08 |
| BCST. perseverative errors | 1.14 (0.88) | 0.2 | 2.61 (2.61) | 0.006 | -0.11 (0.97) | 0.91 | -0.375 ( -0.37 ) | 0.82 | -1.85 (1.23) | 0.13 | 4.432 (1.96) | 0.03 |
